# Complications after Surgical Procedures in Patients with Cardiac Implantable Electronic Devices: Results of a Prospective Registry

**DOI:** 10.5935/abc.20160129

**Published:** 2016-09

**Authors:** Katia Regina da Silva, Caio Marcos de Moraes Albertini, Elizabeth Sartori Crevelari, Eduardo Infante Januzzi de Carvalho, Alfredo Inácio Fiorelli, Martino Martinelli Filho, Roberto Costa

**Affiliations:** Instituto do Coração (InCor) do Hospital das Clínicas da Faculdade de Medicina da Universidade de São Paulo (HCFMUSP), São Paulo, SP - Brazil

**Keywords:** Pacemaker, Artificial, Surgery/complications, Intraoperative Complications/mortality, Defibrillators, Implantable

## Abstract

**Background:**

Complications after surgical procedures in patients with cardiac implantable electronic devices (CIED) are an emerging problem due to an increasing number of such procedures and aging of the population, which consequently increases the frequency of comorbidities.

**Objective:**

To identify the rates of postoperative complications, mortality, and hospital readmissions, and evaluate the risk factors for the occurrence of these events.

**Methods:**

Prospective and unicentric study that included all individuals undergoing CIED surgical procedures from February to August 2011. The patients were distributed by type of procedure into the following groups: initial implantations (cohort 1), generator exchange (cohort 2), and lead-related procedures (cohort 3). The outcomes were evaluated by an independent committee. Univariate and multivariate analyses assessed the risk factors, and the Kaplan-Meier method was used for survival analysis.

**Results:**

A total of 713 patients were included in the study and distributed as follows: 333 in cohort 1, 304 in cohort 2, and 76 in cohort 3. Postoperative complications were detected in 7.5%, 1.6%, and 11.8% of the patients in cohorts 1, 2, and 3, respectively (p = 0.014). During a 6-month follow-up, there were 58 (8.1%) deaths and 75 (10.5%) hospital readmissions. Predictors of hospital readmission included the use of implantable cardioverter-defibrillators (odds ratio [OR] = 4.2), functional class III­-IV (OR = 1.8), and warfarin administration (OR = 1.9). Predictors of mortality included age over 80 years (OR = 2.4), ventricular dysfunction (OR = 2.2), functional class III-IV (OR = 3.3), and warfarin administration (OR = 2.3).

**Conclusions:**

Postoperative complications, hospital readmissions, and deaths occurred frequently and were strongly related to the type of procedure performed, type of CIED, and severity of the patient's underlying heart disease.

## Introduction

Cardiac implantable electronic devices (CIED), including pacemakers (PM), implantable cardioverter-defibrillators (ICD), and cardiac resynchronization therapy (CRT) without (CRT-P) or with defibrillator (CRT-D) are the main innovations in cardiology in the last decades.^1^ More than 737,000 procedures to implant these devices are performed every year worldwide,^2-4^ with an estimated annual average in Brazil of 35,000 new implants and 15,000 reoperations for device maintenance or treatment of device-related complications.^5^

Despite a large increase in the number of procedures and the complexity of the cardiac devices, surprisingly little is known about the effectiveness and safety of these devices, and their impact on the patients' mortality in Brazil. Recent statistics have reported increased complications rates after surgical procedures in patients with CIED, which have been disproportionately higher than the number of initial device implantations.^6-12^

The main factor associated with the increasing incidence of complications in patients with CIED is the aging of the population requiring conventional PM implants, which, in turn, is strongly associated with an increased rate of comorbidities and higher rates of hospital readmissions and mortality.^6-11^ Similarly, the incorporation of ICD and CRT as therapeutic modalities of artificial cardiac pacing has brought a new challenge to this field, since most candidates for these implant devices are patients with severe left ventricular dysfunction often refractory to pharmacological treatment for heart failure.^13-16^ Other factors also justifying the increasing number of complications include procedures to extract old leads, which carry a high surgical risk, and treatment of patients with CIED-related infections, who are often severely septicemic.^6,7,17^

In this study, we implemented a prospective registry gathering data from clinical practice with the purpose of (1) identifying the rates of complications, hospital readmissions, and perioperative mortality within the first 6 months of clinical follow-up, and (2) evaluate the risk factors associated with the occurrence of these events. These data are intended to modify routine protocols in order to prevent and treat these events at an early stage.

## Methods

### Study design and population

The CIED Registry was a single-center prospective study conducted in a hospital providing advanced care. The study was approved by our institution's Research Ethics Committee, and the study participants signed a free and informed consent form.

We included all consecutive patients undergoing any type of surgical procedure involving artificial and permanent cardiac pacing between February and August 2011. The surgical procedures were performed by attending physicians, residents in cardiovascular surgery, and cardiologists undergoing training in artificial cardiac pacing.

All patients were followed up for 6 months after surgery through routine outpatient visits or, when attending other services, through telephone contact.

### Study outcomes

The outcomes evaluated in the study included (1) intraoperative and immediate postoperative complications, or complications within the first 6 months of clinical follow-up; (2) the need for hospital readmissions; and (3) mortality from any cause.

The complications were characterized as (1) major, when life-threatening or requiring surgical reintervention for correction; and (2) minor, when suitable for treatment on an outpatient basis, involving device reprogramming, or requiring exclusive clinical observation. All major complications, hospital readmissions, and deaths were evaluated by an independent expert committee.

### Study dynamics

**Figure 1:**
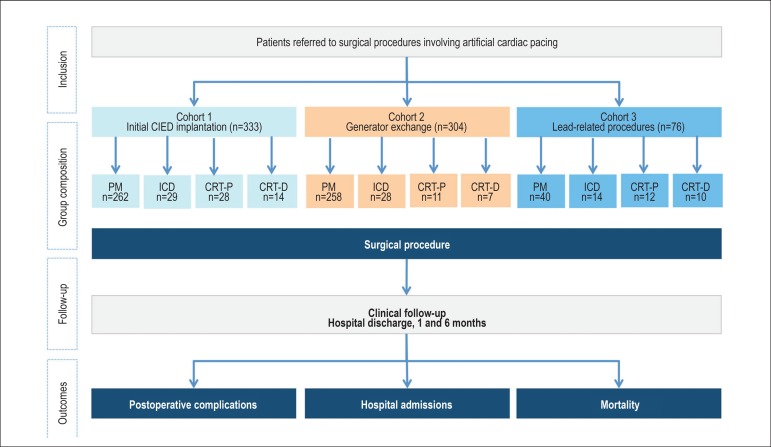
Phases of the study and composition of the studied population. ICD: implantable cardioverter-defibrillator; CIED: cardiac implantable electronic device; PM: pacemaker; CRT: cardiac resynchronization therapy; CRT-D: cardiac resynchronization therapy associated with ICD; CRT-P: cardiac resynchronization therapy alone.

We collected data at four distinct moments: immediately before surgery (immediate preoperative), at hospital discharge, and at 30 days and 6 months after surgery. The figure 1 shows the main phases of the study and the composition of the studied population.

In the immediate preoperative period, we collected demographic and clinical data prior to the CIED implantation, as well as information related to the clinical conditions of the patients upon collection of the data. When available, echocardiographic data were collected to determine the patients' ventricular function. We estimated the patients' left ventricular ejection fraction (LVEF) with the Teicholz method or, preferably, the Simpson method, considering as normal those values above 0.55.

In the assessments performed in the postoperative period and upon hospital discharge, we prioritized the evaluation of complications related to the surgical procedure, clinical complications arising from deterioration of the existing heart disease, and problems directly related to the CIED.

### Electronic collection and management of the data

The data were stored in a database developed in the REDCap (Research Electronic Data Capture) software,^18^ which is hosted in our institution's server.^19^

### Studied variables

We analyzed the following independent variables potentially associated with a risk of occurrence of the studied outcomes: demographic data, preoperative baseline clinical data, type of CIED, and type of procedure performed.

To improve our understanding of the severity of the procedures performed, we grouped the patients into three distinct cohorts: (1) initial implantation of conventional PM, ICD, CRT-P, or CRT-D; (2) change of pulse generators or procedures limited to the pulse generator pocket, characterized in this study as low-risk reoperations; and (3) reoperations involving previously implanted leads, such as lead extraction or upgrade procedures, characterized as high-risk reoperations.

#### Statistical analysis

The data were electronically exported to Excel (Microsoft Excel) spreadsheets and analyzed with SAS (Statistical Analysis System), SPSS (Statistical Package for the Social Sciences), and RStudio.

Quantitative variables are described as mean and standard deviation and qualitative variables as absolute and relative frequencies.

The association of independent variables with the occurrence of the evaluated outcomes was analyzed with chi-square or Fisher exact test. Differences in distribution of quantitative numerical variables according to the occurrence of outcomes (group with and without complications) were evaluated with Student's *t* test. We used multivariate logistic regression with the stepwise variable selection to evaluate independent risk factors, including those variables with associations with a p value ≤ 0.10 in the univariate analysis. Based on the logistic regression model, we estimated the magnitude of the effect of the variables included in the final model by calculating the odds ratios (OR) and their respective 95% confidence intervals (CI). The probability of survival and hospital readmission-free survival were estimated by the Kaplan-Meier method. We adopted a significance level of 5% in the statistical tests.

## Results

Over the 6-month inclusion period, 713 patients underwent surgical procedures comprising 333 (46.7%) initial implantations, 304 (42.6%) low-risk reoperations, and 76 (10.7%) high-risk reoperations. The baseline characteristics of the patients are described in Table 1.

**Table 1:** Demographic and baseline clinical characteristics

Demographic and Baseline Clinical Characteristics	
Male gender, n (%)	367 (51.5)
**Age**	
Mean ± SD (years)	64.5 ± 18.7
Range	4 days to 98.6 years
**Baseline heart disease, n (%)**	
Without structural heart disease	412 (57.8)
Chagasic cardiomyopathy	87 (12.2)
Ischemic cardiomyopathy	59 (8.3)
Nonischemic cardiomyopathy	76 (10.7)
Congenital heart defect	8 (1.1%)
Others	42 (5.9)
Information not available	29 (4.1)
**Associated comorbidities, n (%)**	
None	40 (5.6)
Only one	184 (25.8)
Two	171 (24.0)
Three	251 (35.2)
Four	67 (9.4)
**Functional class (NYHA), n (%)**	
I	368 (51.6)
II	237 (33.2)
III	100 (14.0)
IV	8 (1.1)
Presence of atrial fibrillation	9 (1.3%)
Use of oral anticoagulants	97 (13.6)
Use of antiplatelet agents	277 (38.8)
**LVEF, n (%)**	
Severe dysfunction (LVEF < 40)	166 (23.3)
Moderate dysfunction (LVEF ≥ 40 < 55)	103 (14.4)
Normal ventricular function (LVEF ≥ 55)	355 (49.8)
Information not available	89 (12.5)

SD: standard deviation; LVEF: left ventricular ejection fraction; NYHA: New York Heart Association.

The patients were aged 4 days to 98.6 years with a median of 67.9 years. There was a slight predominance of males (51.5% of the cases). Most (66.4%) patients had no other heart disease apart from the underlying heart rate disturbance. Cardiomyopathy was diagnosed in 31.2% of the patients and was attributed to Chagas disease, or to idiopathic or ischemic causes. Only 1.1% of the patients presented a structural congenital heart disease.

The baseline assessment showed that most patients were oligosymptomatic in terms of manifestations of heart failure: 51.6% were in New York Heart Association (NYHA) functional class (FC) I, 33.2% in FC II, 14.0% in FC III, and 1.1% in FC IV.

Most patients presented with one or more comorbidities: 25.8% of the patients had one comorbidity, while 24.0% and 44.6% had two and three comorbidities, respectively. Only 5.6% of the patients had no other associated disease.

A total of 87.5% of the patients underwent echocardiographic studies. Among these patients, 49.8% had a normal LVEF, while 14.4% had LVEF estimated between 0.40 and 0.55, and 23.3% below 0.40.

During the 6-month follow-up period, adverse events were observed in 204 patients (28.6%). When considered individually, these events comprised 58 (8.1%) deaths, 75 (10.5%) hospital readmissions, 39 (5.5%) major complications, and 165 (23.1%) minor complications (Table 2).

**Table 2:** Distribution of complications according to the type of procedure performed

Complications	All (n= 713)	Initial implantation (n= 333)	Generator exchange (n= 304)	Lead-related procedures (n= 76)	p
Any complication	204 (28.6%)	99 (29.7%)	72 (23.7%)	31 (40.8%)	NS
Major complications	39 (5.5%)	25 (7.5%)	5 (1.6%)	9 (11.8%)	0.014
Lead displacement	19 (2.7%)	14 (4.2%)	-	5 (6.6%)	NS
Cardiac tamponade	1 (0.1%)	1 (0.3%)	-	-	NS
Hemothorax	3 (0.4%)	2 (0.6%)	-	-	NS
Pneumothorax	7 (1.0%)	4 (1.2%)	-	3 (3.9%)	NS
Pocket abscess	3 (0.4%)	1 (0.3%)	2 (0.7%)	-	NS
Endocarditis	2 (0.3%)	1 (0.3%)	1 (0.3%)	-	NS
Lead fracture	1 (0.1%)	-	1 (0.3%)	-	NS
DVT (ipsilateral upper extremity)	3 (0.4%)	2 (0.6%)	-	1 (1.3%)	NS
Minor complications	165 (23.1%)	78 (23.4%)	64 (21.1%)	23 (30.3%)	NS
Phrenic stimulation / muscular	5 (0.7%)	3 (0.9%)	1 (0.3%)	1 (1.3%)	NS
Pace / sense alterations	20 (2.85)	3 (0.9%)	16 (5.3%)	1 (1.3%)	NS
Pocket hematoma	57 (8.0%)	35 (10.5%)	13 (4.3%)	9 (11.8%)	NS
Pocket fluid	43 (6.0%)	14 (4.2%)	21 (6.9%)	8 (10.5%)	NS
Superficial dehiscence	32 (4.5%)	17 (5.1%)	12 (3.9%)	3 (3.9%)	NS
Surface wound infection	7 (1.0%)	5 (1.5%)	1 (0.3%)	1 (1.3%)	NS
Skin scarification	1 (0.1%)	1 (0.3%)	-	-	NS

NS: non-significant; DVT: deep venous thrombosis.

Major complications were significantly more frequent (p = 0.014) in the cohort of patients undergoing high-risk reoperations (11.8%) when compared with those undergoing initial implantations (7.5%), and low-risk reoperations (1.6%). There were no significant differences in rates of minor complications among the three cohorts. The various types of complications observed are listed in Table 3. On univariate analysis, only administration of warfarin (p = 0.030) was identified as a risk factor for major complications, while no risk factors for minor complications were observed.

**Table 3:** Factors influencing the occurrence of complications

**Factors associated with major complications**	**Absence of complication**	**Presence of complication**	**p**
Age	65.1 ± 18.6	64.9 ± 23.4	0.983
Male gender	48.4%	49.3%	0.851
Left ventricular ejection fraction	46.9 ± 21.6	53.2 ± 19.6	0.119
**Type of cardiac device**			
Conventional PM	78.8%	77.6%	0.200
Conventional ICD	10.7%	6.7%	
CRT-D	3.8%	6.7%	
CRT-P	6.7%	8.9%	
**Baseline heart disease**			
Without structural heart disease	66.9%	64.1%	0.273
Chagasic cardiomyopathy	11.4%	18.3%	
Ischemic cardiomyopathy	9.1%	6.9%	
Nonischemic cardiomyopathy	11.4%	9.9%	
**Functional class (NYHA)**			
I - II	85.3%	82.8%	0.807
III - IV	14.7%	17.2%	
Multiple comorbidities	93.8%	97.1%	0.141
Use of antiplatelet agents	38.6%	40.3%	0.713
Use of warfarin	12.2%	19.4%	0.030*
**Factors associated with minor complications**	**Absence of complication**	**Presence of complication**	**p**
Age	63.5 ± 19.8	65.3 ± 16.9	0.318
Male gender	48.6%	47.1%	0.860
Left ventricular ejection fraction	45.5 ± 22.7	48.8 ± 19.4	0.796
**Type of cardiac device**			
Conventional PM	78.9%	70.6%	0.443
Conventional ICD	9.8%	11.8%	
CRT-D	4.4%	5.9%	
CRT-P	6.9%	11.7%	
**Baseline heart disease**			
Without structural heart disease	66.8%	58.8%	0.540
Chagasic cardiomyopathy	12.3%	20.6%	
Ischemic cardiomyopathy	8.5%	11.7%	
Nonischemic cardiomyopathy	11.3%	8.8%	
**Functional class (NYHA)**			
I - II	84.8%	85.3%	0.311
III - IV	15.2%	14.7%	
Multiple comorbidities	94.4%	93.9%	0.707
Use of antiplatelet agents	38.6%	44.1%	0.522
Use of warfarin	13.6%	14.7%	0.798

ICD: implantable cardioverter-defibrillator; PM: pacemaker; CRT: cardiac resynchronization therapy; CRT-D: cardiac resynchronization therapy associated with ICD; CRT-P: cardiac resynchronization therapy alone.

**Figure 2:**
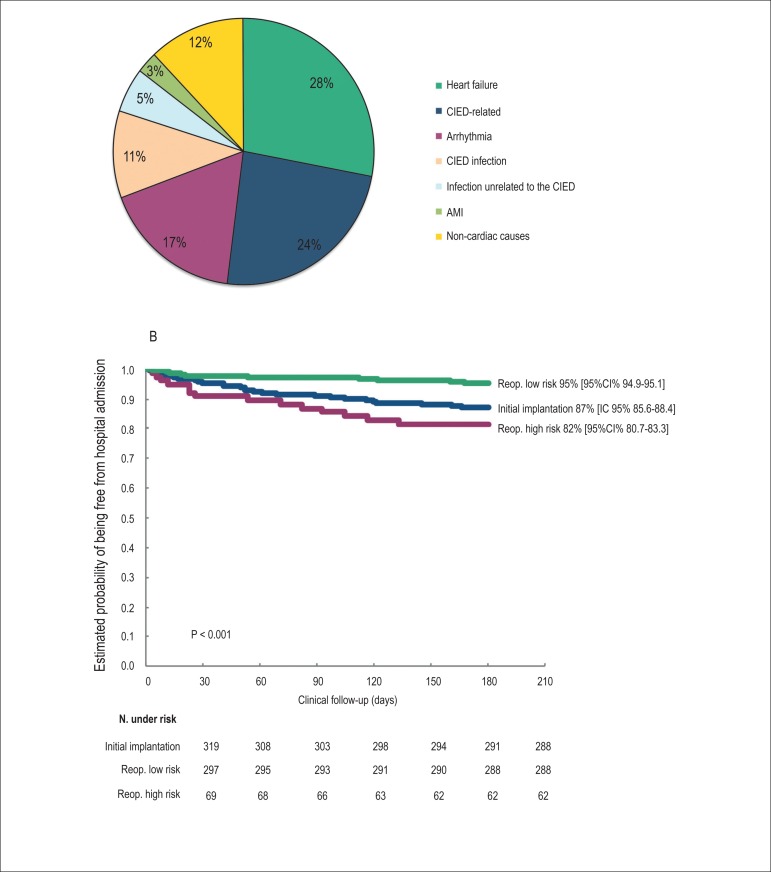
Hospital readmission of patients with CIED during a follow-up period of 6 months after the surgical procedure. (A) Reasons for hospital readmission; (B) Estimated probability of being free from hospital readmission. CIED: cardiac implantable electronic device; AMI: acute myocardial infarction; Reop.: reoperation.

Of the 713 cases studied, 75 (10.5%) required readmission to the hospital within the first 6 months from the operation. In only 26 (3.6%) of these, the readmission was associated with problems related to cardiac pacing (Figure 2A). The expectation of being free from hospital readmission after 6 months of follow-up was 95% (95%CI = 94.9-95.1%), 87% (95%CI = 85.6-88.4%), and 82% (95%CI = 80.7-83.3%) for low-risk reoperations, initial implantation, and high-risk reoperations, respectively (Figure 2B). Figure 2B also shows that hospital readmissions were more frequent in patients undergoing high-risk reoperations (p < 0.001).

**Figure 3:**
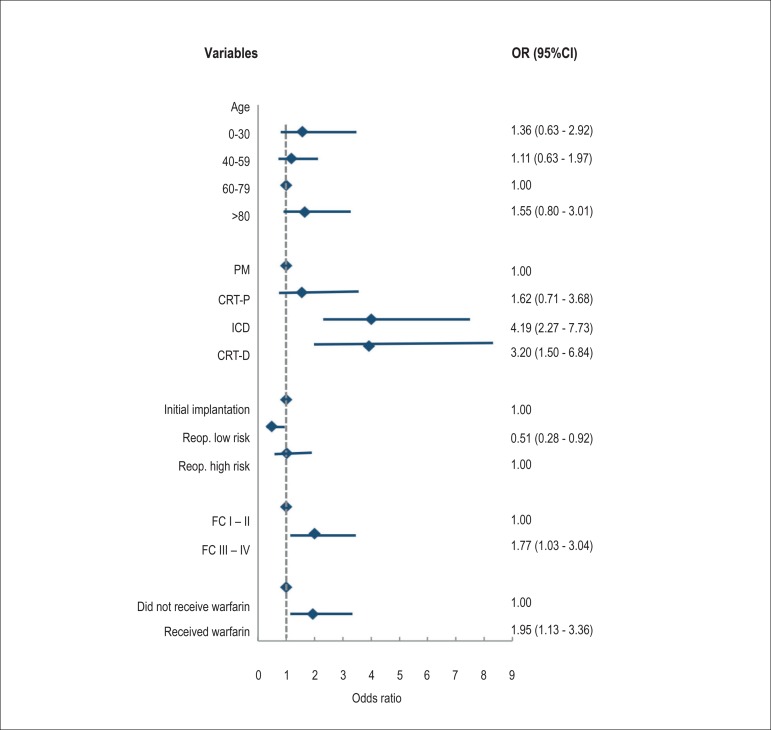
Risk factors for hospital readmission in patients with CIED during a follow-up of 6 months after the surgical procedure. ICD: implantable cardioverterdefibrillator; FC: functional class; PM: pacemaker; Reop. : reoperation; CRT-D: cardiac resynchronization therapy associated with ICD; CRT-P: cardiac resynchronization therapy alone.

We identified the following risk factors for hospital readmission: ICD (OR = 4.19, 95%CI = 2.27-7.73) or CRT-D (OR = 3.20, 95%CI = 1.50-6.84) implants, preoperative NYHA FC III or IV (OR = 1.77, 95%CI = 1.03-3.04), and warfarin administration (OR = 1.95, 95%CI = 1.13-3.36). Patients undergoing low-risk reoperations had half the risk of hospital readmission than the other patients included in the study (Figure 3).

**Figure 4:**
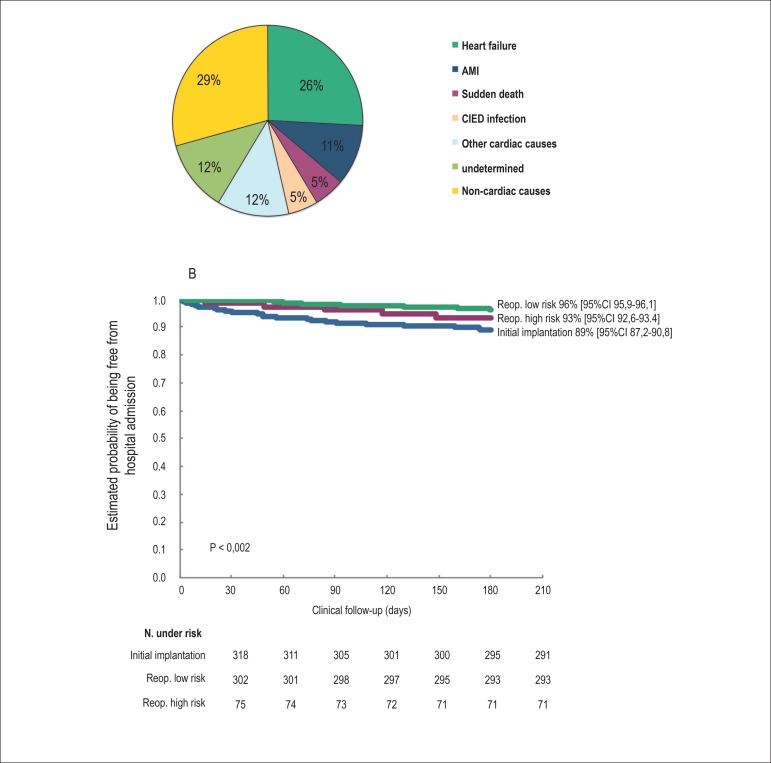
Mortality of patients with CIED during a follow-up of 6 months after the surgical procedure. (A) Mortality causes; (B) Estimated probability of survival. CIED: cardiac implantable electronic device; AMI: acute myocardial infarction; Reop.: reoperation.

The general mortality rate was 8.1% after 6 months of follow-up. Only three deaths were related to problems with the artificial cardiac pacing (Figure 4A). The expected survival rates at 6 months of follow-up were 96% (95%CI = 95.9-96.1%), 93% (95%CI = 92.6-93.4%), and 89% (95%CI = 87.2-90.8%) for low-risk reoperations, high-risk reoperations, and initial implantations, respectively. As shown in Figure 4B, the mortality was higher in the cohort undergoing initial implantation (p = 0.002).

**Figure 5:**
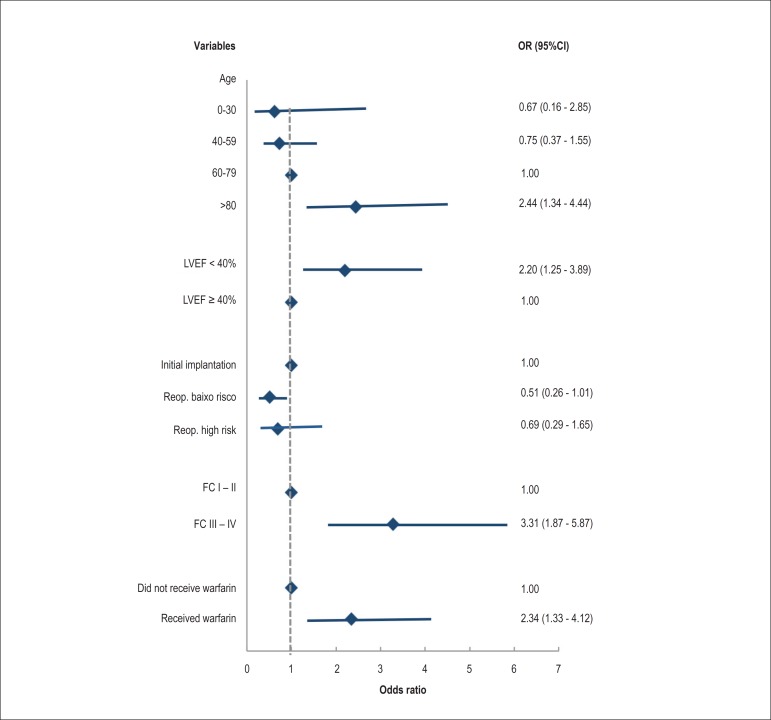
Risk factors for mortality in patients with CIED during a follow-up period of 6 months after the surgical procedure. ICD: implantable cardioverter-defibrillator; FC: functional class; PM: pacemaker; Reop. : reoperation; CRT-D: cardiac resynchronization therapy associated with ICD; CRT-P: cardiac resynchronization therapy alone; LVEF: left ventricular ejection fraction.

We identified the following risk factors for mortality: age over 80 years at surgery (OR = 2.44, 95%CI = 1.34-4.44), LVEF below 0.40 (OR = 2.20, 95%CI = 1.25-3.89), preoperative NYHA FC III or IV (OR = 3.31, 95%CI = 1.87-5.87), and warfarin administration (OR = 2.34, 95%CI = 1.33-4.12). Patients undergoing low-risk reoperations had half the risk of death from any cause when compared with the other patients in the study (Figure 5).

## Discussion

Complications in surgical procedures for implantation or maintenance of CIEDs occur frequently. These complications may result from skin punctures during venous access procedures, handling of vein and cardiac catheters, contamination by infectious agents, anesthetic procedures, or other situations that occur less frequently.^6-12,20^

Despite the fact that complications may occur at random, factors related to their increasing incidence have been described. For example, the experience of the hospital and the surgical team performing the procedure are strongly associated with the number of complications.^21,22^ The type of implanted device and surgery performed also influence the outcome of the procedure.^8-17^ Traditionally, surgeries for implantation of complex devices with a larger number of leads, as well as reoperations involving intravascular handling of the leads, particularly procedures to extract old leads, show a higher risk of complications.^8,9,16,17^

The rates of perioperative and postoperative complications in procedures related to CIED have increased considerably and in disproportion to the number of initial device implantation. Several factors may be related to this fact, including aging of the population, as well as increasing number of comorbidities and prescription of anticoagulants and antiplatelet agents.^6-11^ Another key factor is the incorporation into artificial cardiac pacing of cardioverter-defibrillators and CRT devices, which are mostly used to treat patients with severe left ventricular dysfunction.^13-16,23,24^

The present study identified a high rate of intraoperative and postoperative complications, although these complications were mostly minor in nature and not life-threatening or requiring intervention or hospital readmission for their management. These minor complications occurred at random and were unrelated to the type of procedure performed. On the other hand, major complications that were life-threatening or required reintervention or hospital readmission were more frequent in initial implantations (7.5%), and significantly more frequently in high-risk reoperations (11.8%). These rates, although consistent, were lower than those of major complications reported in the REPLACE Registry, which ranged from 4.0% to 15.3% in patients undergoing generator exchange and upgrade procedures, respectively.^8^

Despite the high rate (10.5%) of hospital readmissions within 6 months from the surgical procedure, this rate was higher in patients undergoing high-risk reoperations, followed by those undergoing initial implantation. We also detected risk factors for this occurrence, mostly related to the severity of the heart disease, such as the requirement of any type of ICD or oral anticoagulant therapy, and preoperative NYHA FC III or IV. Data from the Danish Registry^9^ and the Medicare program^21^ have confirmed a higher morbidity rate with ICD alone or associated with CRT when compared with that with other devices.

Despite the high mortality rate from all causes observed in the same period (8.1%), this event was rarely related to the surgical procedure, but rather to the severity of the disease itself. We observed a higher risk of mortality among patients who were either octogenarians, had severely decreased ventricular function or symptomatic heart failure, or received oral anticoagulant therapy. These rates are consistent with the mortality rates for heart failure reported in the Framingham Heart Study (10% in 30 days and 20-30% in 1 year),^24^ as well as the annual mortality rates of 9% and 12% described in the CARE-HF^25^ and COMPANION^26^ studies, respectively.

On the other hand, patients who underwent procedures to exchange pulse generators alone or other procedures that did not involve intravascular handling had significantly lower risks of death, hospital readmission, or complications than the patients in the other two cohorts. The fact that the majority of these patients had their procedures scheduled electively may have been crucial to their better outcomes.

Literature studies about complications and mortality in patients with CIED are mainly based on secondary analyses of randomized clinical trials or observational studies with limited sample sizes. As far as we know, the sample of this prospective registry is the largest to assess postoperative outcomes in patients with CIED in a single cardiology center in Brazil. Another aspect to be noted is that the data presented in this study reflect a picture of real-world clinical practice, since the analysis included all patients seen during a limited period of time, regardless of age, medical condition or surgical procedure, thus avoiding selection biases that could have invalidated a generalization of the results.

### Limitations of the study

This study presents some limitations that must be considered in the interpretation of the results. Although the study included a representative sample, it reflects the care practices of a single cardiology hospital in the country, which is considered a reference center providing advanced artificial cardiac pacing therapies and a cardiology training center. Since this study was not designed to assess the effects of each surgeon's experience level and/or the volume of procedures performed individually, we are unable to claim that the surgeons' positions on the learning curve influenced the higher risk of intraoperative complications. The possibility of such association will be assessed in future studies conducted at our institution. Finally, a long-term follow-up of this population is especially important to provide more robust evidence about possible adverse events that occur at later stages of care of patients with CIED, which are often underreported.

## Conclusions

We conclude that adverse perioperative and postoperative events were frequent in the studied population. These events were strongly related to the type of procedure performed, type of device implanted, and, mainly, to the severity of the patient's underlying heart disease. We identified risk factors for mortality and hospital readmission, confirming that serious events occur in older patients and in those with more advanced cardiomyopathy.

The findings of this study confirm a need for specific care protocols to follow-up patients at a higher risk of presenting serious events.
